# Editorial: Ankylosing Spondylitis and Related Immune-Mediated Disorders

**DOI:** 10.3389/fimmu.2019.01232

**Published:** 2019-06-04

**Authors:** Maria Teresa Fiorillo, Nigil Haroon, Francesco Ciccia, Maxime Breban

**Affiliations:** ^1^Department of Biology and Biotechnology “Charles Darwin”, Sapienza University of Rome, Rome, Italy; ^2^Department of Medicine, University of Toronto, University Health Network, Kremibil Research Institute, Toronto, ON, Canada; ^3^Department of Precision Medicine, University della Campania L. Vanvitelli, Naples, Italy; ^4^UMR 1173 Inserm/University of Versailles-Saint-Quentin-University of Paris-Saclay, Montigny-le-Bretonneux, France; ^5^Rheumatology Division, Ambroise Paré Hospital, Boulogne-Billancourt, France; ^6^INFLAMEX, Laboratoire d'Excellence, Université Paris Diderot, Sorbonne Paris Cité, Paris, France

**Keywords:** ankylosing spondylitis, HLA-B27 alleles, ERAP, functional genomics, enthesitis, anti-TNF therapy, biomarkers, anti-CD74 autoantibodies

Ankylosing spondylitis (AS) and the related spondyloarthritis (SpA) have become an extremely productive field in immunogenetic research over the last several years, justifying an update. This timely special issue will thoroughly cover the strong heritability of SpA by tackling the explosion of new genetic discoveries in SpA, paving the way for functional studies and also interrogating the striking but still mysterious role of HLA-B27 in disease susceptibility (1).

In the exhaustive review by Costantino et al. the role of genetic factors in SpA pathogenesis is discussed. The association of SpA with HLA-B27 and other MHC/non-MHC genes is reviewed in detail. This is followed by a section dealing with the functional studies exploring the pathogenic role of these novel genetic discoveries. Finally, in the section on the clinical relevance, the potential diagnostic, and prognostic utility of these genetic markers is outlined.

More than 100 genes have been identified to contribute to AS susceptibility even though the mechanisms underlying most of the associations are unknown. Vecellio et al. start their review by discussing the role of HLA-B27, aminopeptidases, and IL-23R in influencing AS susceptibility. A particularly interesting section is dedicated to the analysis of the functional consequences of the genetic association of AS with TBX21 and RUNX3, two transcription factors relevant for tuning the functions of multiple immune cell types.

The article by López de Castro is focused on the contribution given by the immunopeptidomics to understand how the Endoplasmic Reticulum Aminopeptidases ERAP1 and 2, major risk factors for AS and other immune-mediated disorders, shape the peptidomes of the related disease-associated HLA-I molecules. Despite the advances in the structural, biochemical, and functional knowledge of ERAP1 and 2, there is an emerging need to define the influence of ERAP1 polymorphisms and ERAP2 expression in shaping the constitutive HLA-I-bound peptidomes expressed in live cells and eventually involved in disease pathogenesis.

The perspective by Paladini et al. stems from the observation that the ancestral HLA-B^*^2705 allele and the derived B27 subtypes show an opposite gradient in the worldwide distribution and includes into the scheme the ERAP variants known to be associated with AS. Of note, those variants lowering ERAP2 gene expression, have a distribution similar to that of the alternative HLA-B27 subtypes. Hence, focusing on Sardinia island, the authors made the hypothesis that these genes, both involved in AS pathogenesis, co-evolved under the environmental pressure by pathogens such as *Plasmodium falciparum*, and that the disease results as a trade-off of these interconnections.

A very important matter for any chronic disease concerns the understanding of early events predating full disease establishment, since this may open the door for preventive care. Accordingly, Watad et al. conceptualized the initial stages of AS which link the skeletal mechanical stress and the tissue microdamage to an abnormal immunological response and exuberant repair process. These are indicated as major events which cause enthesitis and osteitis in the sacroiliac joints and are followed by post-inflammatory chronic new bone formation in genetically predisposed subjects. Particular emphasis is given to the gut inflammation and to the role of altered microbiota which are linked to joint manifestations through a still undefined immunological axis. The role of entheseal resident cells and pivotal cytokines for disease initiation is also discussed.

A crucial topic that emerges from this article collection is the promise of novel predictive biomarkers, particularly but not exclusively blood-derived, since the gut might also provide an interesting contribution. This will be of interest not only to help diagnosis but also to predict prognosis and therapeutic success, notably with the widely used anti-TNF therapies as well as to personalize treatment options. The review by Maksymowych comprehensively covers the field by a systematic overview of the recent studies focused on the findings of new candidate biomarkers (autoantibodies, antibodies to gut microbiota, inflammatory markers, miRNAs, disease-specific metabolites, cytokines, adipokines, tissue turnover protein fragments). It emerges that CRP remains the only reliable predictive biomarker of disease progression while fecal calprotectin is a consistent indicator of gut inflammation in Axial SpA.

Historically, AS has been considered the prototype of seronegative arthritis. Recent evidence indicating the presence of anti-CD74 autoantibodies in patients with AS, suggests that the concept of seronegativity in AS should be reviewed. CD74 is the receptor of the macrophage migration inhibitory factor (MIF) which is involved in the pathogenesis of AS and mediates its proinflammatory function. Liu et al., in their exhaustive review summarize the recent evidence on the role of anti-CD74 autoantibodies in AS, also presenting their data which discard these autoantibodies as good biomarker for SpA diagnosis in Asian population.

Menegatti et al. explore the important area of personalized medicine discussing different treatment modalities in spondyloarthritis and drawing parallels from treatment of Rheumatoid Arthritis patients. Starting with the history of the usage of TNF inhibitors in rheumatology, they extend the review to their role in spondyloarthritis. The current literature on predicting which patients are likely to respond to TNF inhibitors is discussed. The review concludes with a description of the more recent drugs that block type 3 immunity.

Many unanswered questions dictate the priorities that need to be addressed. Diligent integration of susceptibility gene polymorphisms with cell/tissue expression data is needed to understand their functional consequence. Epigenetics is an emerging approach in the field of SpA that will require more investment. Well-characterized cohorts of patients and pre-clinical models, as well as up-to-date techniques are required to improve the discovery of efficient biomarkers. From these novel and emerging approaches suitable targets for curative or, even better, preventive interventions are likely to emerge. This is what we need at the end.

## Author Contributions

All authors listed have made a substantial, direct and intellectual contribution to the work, and approved it for publication.

### Conflict of Interest Statement

The authors declare that the research was conducted in the absence of any commercial or financial relationships that could be construed as a potential conflict of interest.
